# Supplementary material for: Sexual health beliefs and prevention of sexually transmitted infections among cisgender women sex workers in Colombia
Source: PLoS One. 2024 Jun 12;19(6):e0305293. doi: 10.1371/journal.pone.0305293 (PMC11168633; doi:10.1371/journal.pone.0305293)
Supplement: Attachment — Submitted filename: Response to Reviewers.docx [file pone.0305293.s001.docx]

**Answers to all points raised by the academic editor and reviewers of the manuscript.**

We express our sincere thanks to the Academic Editor and reviewers for their dedicated time and effort in reviewing our manuscript entitled “*Sexual health beliefs and prevention of sexually transmitted infections among cisgender women sex workers in Colombia,*” submitted for consideration by the journal PLOS ONE under reference number PONE-D-24-05771. We have thoroughly evaluated each of the recommendations provided and have incorporated them in their entirety, as they are of great relevance to guide the improvement of our manuscript. Below each comment from the academic editor and reviewers, you will find our answers, identified with the subheading "**Answer #**" underlined and highlighted in blue and bold and the text of the answer in blue.

PONE-D-24-05771

Sexual health beliefs and prevention of sexually transmitted infections among cisgender women sex workers in Colombia.

PLOS ONE

Dear Dr. Goberna-Tricas,

Thank you for submitting your manuscript to PLOS ONE. After careful consideration, we feel that it has merit but does not fully meet PLOS ONE’s publication criteria as it currently stands. Therefore, we invite you to submit a revised version of the manuscript that addresses the points raised during the review process.

Congratulations with interesting research and a well written article. Minor changes were recommended by the reviewers. Kindly, use the feedback to enrich your article.

Consider adding Recommendations.

It is advisable not to use names and rather use pseudonyms or numbers.

The authors may indicate clearly to the readers the reasons for excluding trans-gender SW.

Please submit your revised manuscript by May 25 2024 11:59PM. If you will need more time than this to complete your revisions, please reply to this message or contact the journal office at [plosone@plos.org](mailto:plosone@plos.org). When you're ready to submit your revision, log on to https://www.editorialmanager.com/pone/ and select the 'Submissions Needing Revision' folder to locate your manuscript file.

Please include the following items when submitting your revised manuscript:

- A rebuttal letter that responds to each point raised by the academic editor and reviewer(s). You should upload this letter as a separate file labeled 'Response to Reviewers'.
- A marked-up copy of your manuscript that highlights changes made to the original version. You should upload this as a separate file labeled 'Revised Manuscript with Track Changes'.
- An unmarked version of your revised paper without tracked changes. You should upload this as a separate file labeled 'Manuscript'.

If you would like to make changes to your financial disclosure, please include your updated statement in your cover letter. Guidelines for resubmitting your figure files are available below the reviewer comments at the end of this letter.

If applicable, we recommend that you deposit your laboratory protocols in protocols.io to enhance the reproducibility of your results. Protocols.io assigns your protocol its own identifier (DOI) so that it can be cited independently in the future. For instructions see: <https://journals.plos.org/plosone/s/submission-guidelines#loc-laboratory-protocols>.

Additionally, PLOS ONE offers an option for publishing peer-reviewed Lab Protocol articles, which describe protocols hosted on protocols.io. Read more information on sharing protocols at <https://plos.org/protocols?utm_medium=editorial-email&utm_source=authorletters&utm_campaign=protocols>.

We look forward to receiving your revised manuscript.

Kind regards,

Deidre Pretorius, PhD
Academic Editor
PLOS ONE

**Comment 1:**

Journal Requirements:

When submitting your revision, we need you to address these additional requirements.

1. Please ensure that your manuscript meets PLOS ONE's style requirements, including those for file naming. The PLOS ONE style templates can be found at 
https://journals.plos.org/plosone/s/file?id=wjVg/PLOSOne_formatting_sample_main_body.pdf and 
<https://journals.plos.org/plosone/s/file?id=ba62/PLOSOne_formatting_sample_title_authors_affiliations.pdf>

**Answer 1:**

We have ensured that the revised version of our manuscript follows all PLOS ONE style requirements based on the journal's style templates. We have also applied the file domination requirements to upload it to the journal's online submission system.

Tables 1 and 2, previously included as supplementary information, have been relocated within the manuscript, following PLOS ONE style recommendations. Table 1, which describes the participants' sociodemographic characteristics, is now placed between lines 243 and 244, while Table 2, which details the interview script, is placed between lines 274 and 275 of the manuscript.

Figures 1, 2, and 3 were removed from the supporting information and instead were loaded into the submission system as figures. The files were named as follows:

Fig 1.tif

Fig 2.tif

Fig 3.tif

**Comment 2:**

2. Please provide additional details regarding participant consent. In the ethics statement in the Methods and online submission information, please ensure that you have specified what type you obtained (for instance, written or verbal, and if verbal, how it was documented and witnessed). If your study included minors, state whether you obtained consent from parents or guardians. If the need for consent was waived by the ethics committee, please include this information.

**Answer 2:**

We have ensured a detailed description of the ethical considerations of our research, both in the methods section and in the journal's online submission system. In these sections, we have specified that the Bioethics Commission of the University of Barcelona, Spain, approved the conduct of the study. We invited the 34 cisgender women sex workers to participate, providing them with understandable verbal and written information, and all women gave their consent to participate in the research voluntarily. Finally, we clarify that the textual quotations supporting the themes of our manuscript are de-identified and accompanied by women's pseudonyms to safeguard the anonymity of the participants.

**Comment 3:**

3. Thank you for stating the following financial disclosure:

"JGT
Grant PID2022-140179OB-I00 funded by MCIN/AEI/ 10.13039/501100011033 and, “ERDF A way of making Europe”. 
(Ministerio de Ciencia, Innovación y Univesidades. Agencia Estatal de Investigación).

LAPS
Scholarship for doctoral study abroad, call 885 of 2020 (reference number 20211730037901), granted by the Ministry of Science, Technology and Innovation of Colombia (Minciencias)"

Please state what role the funders took in the study. If the funders had no role, please state: "The funders had no role in study design, data collection and analysis, decision to publish, or preparation of the manuscript."

If this statement is not correct you must amend it as needed. 
Please include this amended Role of Funder statement in your cover letter; we will change the online submission form on your behalf.

**Answer 3:**

Regarding the financial disclosure statement provided in the journal's online submission system, we assure you that the funders had no role in the study design, data collection and analysis, decision to publish, or manuscript preparation.

**Comment 4:**

4. We note that you have indicated that there are restrictions to data sharing for this study. PLOS only allows data to be available upon request if there are legal or ethical restrictions on sharing data publicly. For more information on unacceptable data access restrictions, please see http://journals.plos.org/plosone/s/data-availability#loc-unacceptable-data-access-restrictions.

Before we proceed with your manuscript, please address the following prompts:

a) If there are ethical or legal restrictions on sharing a de-identified data set, please explain them in detail (e.g., data contain potentially identifying or sensitive patient information, data are owned by a third-party organization, etc.) and who has imposed them (e.g., a Research Ethics Committee or Institutional Review Board, etc.). Please also provide contact information for a data access committee, ethics committee, or other institutional body to which data requests may be sent.

b) If there are no restrictions, please upload the minimal anonymized data set necessary to replicate your study findings to a stable, public repository and provide us with the relevant URLs, DOIs, or accession numbers. For a list of recommended repositories, please see
https://journals.plos.org/plosone/s/recommended-repositories. You also have the option of uploading the data as Supporting Information files, but we would recommend depositing data directly to a data repository if possible.

We will update your Data Availability statement on your behalf to reflect the information you provide.

**Answer 4:**

**Data Availability:** The data is related to qualitative interviews with sexual workers is in Spanish language and is considered sensible and confidential information. We are unable to share these data publically because of restrictions by the Bioethics Commission of University of Barcelona as participants did not consent to sharing of their data outside of the study team.

For obtaining information regarding this research and data, the designated entity to contact is the “Secretaría General de la Universidad de Barcelona” (Gran Via de les Corts Catalanes, 585, 08007 Barcelona), email address: secretaria.general@ub.edu.

**Comment 5:**

5. Please ensure that you refer to Figures 1-3 in your text as, if accepted, production will need this reference to link the reader to the figure.

**Answer 5:**

We have verified and ensured that Figures 1-3 have been correctly referenced in the manuscript as Fig 1 (cited in line 208 and page 10), Fig 2 (cited in line 286 and page 15), and Fig 3 (cited in line 316 and page 17). This action will ensure that the figures can be effectively linked in the manuscript's text.

**Comment 6:**

6. Please include a separate caption for each figure in your manuscript.

**Answer 6:**

We have incorporated legends for each figure just after the paragraph in which they are first cited, following PLOS ONE guidelines, remaining as follows:

Fig 1. Strategies for inviting participants. (line 209 and page 10)

Fig 2. Braun and Clarke's reflexive thematic analysis. (line 287 and page 15)

Fig 3. Themes related to sexual health beliefs. (line 319 and page 17)

**Comment 7:**

7. Please review your reference list to ensure that it is complete and correct. If you have cited papers that have been retracted, please include the rationale for doing so in the manuscript text, or remove these references and replace them with relevant current references. Any changes to the reference list should be mentioned in the rebuttal letter that accompanies your revised manuscript. If you need to cite a retracted article, indicate the article’s retracted status in the References list and also include a citation and full reference for the retraction notice.

**Answer 7:**

We thoroughly check our reference list for completeness and accuracy. We will ensure that all references cited in the text are correctly listed and meet the style and formatting standards required by PLOS ONE. It has not been necessary to delete any references. Finally, we have added two new references from two scientific articles cited in the explanation of why the transgender sex worker population was excluded from the study. These references are numbers 40 and 41, located between lines 810 and 816 on page 39 of the manuscript.

**Comment 8:**

Additional Editor Comments:

Reviewer 1: Sexual health beliefs and prevention strategies among cisgender women sex workers are multifaceted and influenced by various factors including cultural, social, economic, and individual circumstances.

Consider adding Recommendations
E.g. Empowering cisgender women sex workers to advocate for their sexual health and well-being. This will includes promoting their right to access healthcare services without discrimination, providing resources for self-care and prevention, and supporting peer-led initiatives that promote solidarity and mutual support within cisgender sex worker communities.

**Answer 8:**

We consider reviewer one's suggestion to add recommendations to the manuscript to be very pertinent. Therefore, we have included two specific recommendations derived from our study's results in the conclusions section. These recommendations are found between lines 655 and 663 on page 32 of the manuscript.

**Comment 9:**

Reviewer 2: Very good and relevant article. However, I am a little bit concerned about the fact that the authors are mentioning the names of the SW interviewed. As a reviewer, I may have direct access to the model of the consent forms signed by the participants. For this reason, author (s) may indicate that the participants allowed them to mention their names to avoid a break of confidentiality. The authors may indicate clearly to the readers the reasons for excluding trans-gender SW.

**Answer 9:**

We want to clarify to reviewer two that the names associated with each textual quote from the cisgender women sex workers are pseudonyms, not their real names. This measure was implemented to protect the participants' anonymity, as explained between lines 218 to 219, exactly in the ethical considerations section of the study.

We consider very relevant the need to clearly explain in the manuscript why we have included in our research only cisgender women sex workers and excluded transgender sex workers. Therefore, we added three explanatory paragraphs in the participant selection and characteristics section. These paragraphs are found on page 11, between lines 225 and 240 of the manuscript.

It's important to explicitly state that we recognize the absence of transgender sex workers in our research as a limitation of its scope. We have included this as a fourth limitation in the corresponding section, page 30, between lines 623 and 626, of the manuscript.

[Note: HTML markup is below. Please do not edit.]

Reviewers' comments:

Reviewer's Responses to Questions

**Comments to the Author**

1. Is the manuscript technically sound, and do the data support the conclusions?

The manuscript must describe a technically sound piece of scientific research with data that supports the conclusions. Experiments must have been conducted rigorously, with appropriate controls, replication, and sample sizes. The conclusions must be drawn appropriately based on the data presented.

Reviewer #1: Yes

Reviewer #2: Yes

2. Has the statistical analysis been performed appropriately and rigorously?

Reviewer #1: Yes

Reviewer #2: Yes

3. Have the authors made all data underlying the findings in their manuscript fully available?

The [PLOS Data policy](http://www.plosone.org/static/policies.action#sharing) requires authors to make all data underlying the findings described in their manuscript fully available without restriction, with rare exception (please refer to the Data Availability Statement in the manuscript PDF file). The data should be provided as part of the manuscript or its supporting information, or deposited to a public repository. For example, in addition to summary statistics, the data points behind means, medians and variance measures should be available. If there are restrictions on publicly sharing data—e.g. participant privacy or use of data from a third party—those must be specified.

Reviewer #1: No

Reviewer #2: Yes

4. Is the manuscript presented in an intelligible fashion and written in standard English?

PLOS ONE does not copyedit accepted manuscripts, so the language in submitted articles must be clear, correct, and unambiguous. Any typographical or grammatical errors should be corrected at revision, so please note any specific errors here.

Reviewer #1: Yes

Reviewer #2: Yes

5. Review Comments to the Author

Please use the space provided to explain your answers to the questions above. You may also include additional comments for the author, including concerns about dual publication, research ethics, or publication ethics. (Please upload your review as an attachment if it exceeds 20,000 characters)

Reviewer #1: Well written article of good scientific and social value.

Sexual health beliefs and prevention strategies among cisgender women sex workers are multifaceted and influenced by various factors including cultural, social, economic, and individual circumstances

Consider adding Recommendations
E.g. Empowering cisgender women sex workers to advocate for their sexual health and well being. This will includes promoting their right to access healthcare services without discrimination, providing resources for self care and prevention, and supporting peer-led initiatives that promote solidarity and mutual support within cisgender sex worker communities.

Reviewer #2: Very good and relevant article. However, I am a little bit concerned about the fact that the authors are mentioning the names of the SW interviewed. As a reviewer, I may have direct access to the model of the consent forms signed by the participants. For this reason, author (s) may indicate that the participants allowed them to mention their names to avoid a break of confidentiality. The authors may indicate clearly to the readers the reasons for excluding trans-gender SW.

6. PLOS authors have the option to publish the peer review history of their article ([what does this mean?](https://journals.plos.org/plosone/s/editorial-and-peer-review-process#loc-peer-review-history)). If published, this will include your full peer review and any attached files.

If you choose “no”, your identity will remain anonymous but your review may still be made public.

**Do you want your identity to be public for this peer review?** For information about this choice, including consent withdrawal, please see our [Privacy Policy](https://www.plos.org/privacy-policy).

Reviewer #1: No

Reviewer #2: **Yes:** Roland Mbuyi Tshibeya

[NOTE: If reviewer comments were submitted as an attachment file, they will be attached to this email and accessible via the submission site. Please log into your account, locate the manuscript record, and check for the action link "View Attachments". If this link does not appear, there are no attachment files.]

While revising your submission, please upload your figure files to the Preflight Analysis and Conversion Engine (PACE) digital diagnostic tool, <https://pacev2.apexcovantage.com/>. PACE helps ensure that figures meet PLOS requirements. To use PACE, you must first register as a user. Registration is free. Then, login and navigate to the UPLOAD tab, where you will find detailed instructions on how to use the tool. If you encounter any issues or have any questions when using PACE, please email PLOS at [figures@plos.org](mailto:figures@plos.org). Please note that Supporting Information files do not need this step.
